# Bias in Research Grant Evaluation Has Dire Consequences for Small Universities

**DOI:** 10.1371/journal.pone.0155876

**Published:** 2016-06-03

**Authors:** Dennis L. Murray, Douglas Morris, Claude Lavoie, Peter R. Leavitt, Hugh MacIsaac, Michael E. J. Masson, Marc-Andre Villard

**Affiliations:** 1 Institute of Integrative Conservation Biology, Trent University, Peterborough, ON, K9J 7B8, Canada; 2 Department of Biology, Lakehead University, Thunder Bay, ON, P7B 5E1, Canada; 3 École supérieure d’aménagement du territoire et de développement régional, Université Laval, Québec, QC, G1V 0 A6, Canada; 4 Department of Biology, University of Regina, Regina, SK, S4S 0A2, Canada; 5 Great Lakes Institute for Environmental Research, University of Windsor, Windsor, ON, N9B 3P4, Canada; 6 Department of Psychology, University of Victoria, Victoria, BC, V8W 2Y2, Canada; 7 Département de biologie, Université de Moncton, Moncton, NB, E1A 3E9, Canada; Universidad de Las Palmas de Gran Canaria, SPAIN

## Abstract

Federal funding for basic scientific research is the cornerstone of societal progress, economy, health and well-being. There is a direct relationship between financial investment in science and a nation’s scientific discoveries, making it a priority for governments to distribute public funding appropriately in support of the best science. However, research grant proposal success rate and funding level can be skewed toward certain groups of applicants, and such skew may be driven by systemic bias arising during grant proposal evaluation and scoring. Policies to best redress this problem are not well established. Here, we show that funding success and grant amounts for applications to Canada’s Natural Sciences and Engineering Research Council (NSERC) Discovery Grant program (2011–2014) are consistently lower for applicants from small institutions. This pattern persists across applicant experience levels, is consistent among three criteria used to score grant proposals, and therefore is interpreted as representing systemic bias targeting applicants from small institutions. When current funding success rates are projected forward, forecasts reveal that future science funding at small schools in Canada will decline precipitously in the next decade, if skews are left uncorrected. We show that a recently-adopted pilot program to bolster success by lowering standards for select applicants from small institutions will not erase funding skew, nor will several other post-evaluation corrective measures. Rather, to support objective and robust review of grant applications, it is necessary for research councils to address evaluation skew directly, by adopting procedures such as blind review of research proposals and bibliometric assessment of performance. Such measures will be important in restoring confidence in the objectivity and fairness of science funding decisions. Likewise, small institutions can improve their research success by more strongly supporting productive researchers and developing competitive graduate programming opportunities.

## Introduction

Federal funding for basic scientific research is an important cornerstone of societal progress, economy, sustainability, and health and well-being. A nation’s scientific knowledge, competitiveness and contributions ultimately can be measured by the amount and type of funding provided for research [1–3]. Accordingly, governments prioritize meritorious and strategic distribution of public funding in support of the best science, often in accordance with other national goals to promote diversity and regional wealth distribution. National research councils are responsible for coordinating disbursement of university-based science through competitive granting programs [4]. Although research councils may use different criteria for the evaluation and selection of proposals for funding, most rely on external grant proposal peer-review and expert selection committees to identify researchers and research programs with the most potential to advance knowledge [4, 5]. As a further strategy, research councils can offer different funding programs, or differentially classify proposals from a single competition, to ensure that funding reaches a broad range of applicants, disciplines, and policy objectives. Collectively, these longstanding and widespread measures are designed to facilitate distribution of science research funding that is both fair and reflective of broader societal needs.

A basic tenet of the science grant proposal adjudication process is that proposal reviewers and selection committees assess submitted applications objectively and appropriately [6–8]. To this end, most research councils adopt quality assurance measures such as grant proposal reviewer anonymity, multiple reviews, and selection committee consensus. These procedures usually succeed in identifying flawed or conceptually weak proposals [5, 9]. However, decisions concerning ranking and funding level of more competitive proposals can be markedly subjective and opaque, often leading to questions about the outcome of decisions and the integrity of the adjudication process [7, 8]. Indeed, factors such as applicant gender, research discipline, seniority level, and institutional affiliation, have been raised as potential sources of evaluation skew [7–9]. However, whether skew in these attributes reflects true differences in applicant merit versus systemic bias against certain groups of applicants can be particularly challenging to resolve, especially because many funding decisions are driven by human perception and consensus, which are difficult to quantify [10–12]. Indeed, finding causes underlying skewed evaluation scores and funding decisions is especially challenging [13], leading to recent questions about whether evaluation skew is even as prevalent or as unjustified as was previously thought [14, 15]. Yet, even if funding skew correctly reflects differential merit, it may nonetheless clash with broader policy objectives such as targeted resource distribution or promoting diversity, and thereby be unwanted by research councils. Regardless, unjustified or unwanted grant proposal evaluation skew should be a major concern because it disadvantages individual researchers, their institutions and students, it calls into question integrity of the funding process itself, and it compromises not only fair distribution of resources but also productivity and future discovery [16, 17].

Research councils should ensure the integrity of science research funding by establishing procedures that entrench fairness and transparency in the evaluation process. This means that research councils should prioritize assessment and detection of grant proposal evaluation skew, determine whether skew is a result of systemic bias, and design policies and procedures to best minimize future skew, if skewed funding outcomes are unwanted. It follows that research councils may be further compelled to develop corrective measures to mitigate the impact of uncorrected evaluation skew on funding results. However, to date, research councils have not broadly adopted measures to directly address evaluation skew and systemic bias, nor have such measures been rigorously evaluated in terms of their effectiveness in correcting unwanted skew.

In this paper, we analyze four years of funding levels and success rates for applications submitted to Canada’s Natural Sciences and Engineering Research Council (NSERC) individual research grants (Discovery Grant) program (2011–2014). Since 1978, NSERC has been responsible for adjudicating and dispensing Discovery Grants. Proposals are reviewed by discipline-based evaluation groups that handle applications in established research areas [18]. Our analysis examined whether funding decisions were related to applicant seniority and institutional affiliation, and whether observed patterns of skew against smaller institutions are related to systemic bias during grant proposal evaluation. We projected patterns of funding success through time to quantify how the observed skew would translate to differential funding rates across institution sizes, then assessed how corrective measures might alleviate skew. Because our results likely reflect patterns that apply to a variety of national research councils and funding programs, we conclude by offering general guidelines for addressing evaluation skew and bias in science research grant funding.

## Materials and Methods

### NSERC Discovery Grant program

NSERC is Canada’s primary university-based scientific research funder, and the Discovery Grant program is the cornerstone of basic and applied post-secondary research in the natural and engineering-related sciences. Discovery Grants cover research-related expenses including: 1) salaries and benefits for graduate students, post-doctoral fellows, and technical/professional assistants; 2) equipment or facility expenses including purchase or rental of equipment, operation and maintenance costs, and user fees; 3) research materials and supplies; 4) travel to conferences, field locations, and consultations; 5) dissemination (publication) costs; and 6) any other research-related costs. Discovery Grants do not cover applicant salaries. Discovery Grants support 10,000 researchers and 30,000 trainees at over 40 post-secondary institutions, with an annual budget of $360 million [18]. The Discovery Grant program is distinguished from programs supported by many other national research councils by supporting faculty research programs rather than specific research projects. Overall, grant duration is longer (normally 5 years) and average grant renewal success rates are higher (78%) than those typically seen from other research councils, albeit with smaller mean grant sizes ($32,000 CAD/year) [18, 19].

NSERC Discovery Grant proposals are reviewed using a conference model involving 12 separate discipline-based evaluation groups. The evaluation process consists of solicited external anonymous grant proposal peer-reviews and expert selection committee reviews that evaluate the merits of each application. The Discovery Grant review process was modified in 2010 to include an evaluation process based on three equally-weighted criteria; Excellence of Researcher (EoR), Merit of Proposal (MoP), and Contribution to the Training of Highly Qualified Personnel (HQP). Unlike systems based on bibliometric analysis of EoR, NSERC provides instructions to grant proposal reviewers that are qualitative (S1 Table), making it unlikely that these evaluations are devoid of subjectivity. MoP is based on the scientific merit of a 5-page research program proposal. The HQP criterion is determined by the number and denomination of past and current trainees, their success during the period of mentorship, and their subsequent disposition after degree completion. Each criterion is assessed on a 6-point scale (S1 Table), with each of five expert selection committee members scoring the proposal according to the three criteria. The final score represents the median of the equally-weighted criteria. The composite evaluation score is then used to position the application in one of 16 funding bins that determine funding status and amount awarded. To an extent, evaluation groups are able to adjust scoring to address discipline-specific context and priorities, but normally a minimum grade of ‘Strong’ is needed on all 3 criteria for applicants to receive funding. There may be minor adjustments to the funding level based on whether anticipated funding costs associated with the proposed work deviate from the norm, or if the applicants are early in their career [18]. For Early Career Researchers (ECRs), normally one of the criteria can receive a ‘Moderate’ score for funding to be granted.

### Analysis of Funding Success and Funding Levels

We analyzed 13,526 Discovery Grant proposal review scores made available to us by NSERC. Our primary dataset includes individual funding results for each application received by the Discovery Grant competition (2011–2014); a secondary dataset was comprised of summary statistics for Discovery Grant proposal funding success rates spanning 2004–2015. This primary dataset coincided with the 2^nd^ to 5^th^ years after NSERC adopted the new evaluation system using the aforementioned scoring criteria. Notably, this new model was designed to establish evaluation criteria independently of applicant performance or funding success prior to the most recent granting period. Our data include NSERC’s classification of each applicant into one of three categories: Established researchers are individuals who serve as faculty, adjunct faculty, or emeritus professors at a postsecondary institution. In our dataset, established researchers are further differentiated according to whether they currently held a Discovery Grant and were thus seeking a renewal (ER-R), versus those not currently holding a Discovery Grant (ER-NHG); the latter group is mainly comprised of former ER-Rs or ECRs who have not received a grant renewal. Early Career Researchers are recognized as researchers in the first two years of their first postsecondary position who have not previously held a Discovery Grant [20]. The dataset also includes NSERC’s classification of the size of postsecondary institutions as large, medium or small, based on the total annual value of funds that they receive from NSERC (approximate categories: large > $14 M; medium $4 M—$14 M; small < $4 M). In general, these size categories are comparable to those derived from estimates of total student population size or the percentage of graduate students in the cohort (Table 1). Periodically, NSERC reviews institutional funding status and schools may be reclassified if their performance changes. Although the institution size category is a relatively coarse metric that may limit our ability to infer fine-scale associations between institution size and research productivity, the classification scheme is reproducible among metrics and exhibits reasonably low variation (Table 1).

**Table 1:** Profile of Canadian universities receiving Discovery Grant funding (2015). Student enrolment is available from: https://en.wikipedia.org/wiki/List_of_universities_in_Canada (accessed April 22, 2016) and includes all programs of study. Discovery Grant funding rates are in $ CDN, and means (± SD) are calculated using institutional summaries, which exclude those with <5 applicants per category [18]. Institution size category corresponds to NSERC binning categories. Applicant types are ER-R (established researcher applying for renewal), ER-NHG (established researcher not holding grant), and ECR (early-career researcher).

	Large	Medium	Small
No. universities	20	10	39
No. students	32361 (15478)	25161 (17953)	8272 (7235)
Percent graduates	18.8	12.7	8.9
ER-R success rate	83.7 (5.2)	76.5 (13.2)	69.1 (15.3)
ER-NHG success rate	41.8 (11.5)	35.4 (13.0)	23.2 (19.0)
ECR success rate	64.5 (18.5)	75.4 (9.5)	58.1 (8.5)
ER-R grant size	35888 (3461)	29891 (3810)	31654 (7649)
ER-NHG grant size	27125 (3088)	24478 (3927)	22643 (3491)
ECR grant size	26899 (2333)	25650 (1594)	24003 (1518)
ER-R funding (total)	1964772 (1079539)	571474 (175533)	194123 (89170)
ER-NHG funding (total)	412824 (309416)	140222 (57619)	52643 (37303)
ECR funding (total)	338390 (217699)	207800 (73168)	88260 (16923)
Total funding	2930533 (1534981)	1060254 (138258)	302927 (7001)

Our primary data include whether the application was funded, the funding bin it was assigned to, and individual scores on each of the evaluation criteria. Data on applicant gender, NSERC evaluation group, institutional identity, and whether the applicant was seeking a first grant renewal, were requested but not made available.

### Data analysis and forecasting

For our initial analysis, we evaluated grant proposal funding success rates, funding levels, and individual evaluation scores for established researchers (ER-R and ER-NHG combined) and ECRs across the three sizes of institutions. We used ordinary logistic regression [21] to determine patterns of funding success for established researchers and ECRs based on a series of *a priori* models; differences in funding bins and funding levels and scores on each of the three evaluation criteria were examined using ordered logistic regression [22], which allowed us to rank evaluation scores while retaining a logistic framework for analysis. Because our analyses involved separate model selection exercises for each criterion, for simplicity we report odds ratios for the best-fit models only. We also describe longer-term trends in grant proposal funding success rates (2004–2015) from data obtained through NSERC records (M. Masson, *unpubl*.).

We forecasted Discovery Grant funding success according to institution size as the proportional (%) change in number of current grant holders through a 10-year period, corresponding to 2 complete grant renewal cycles. We estimated baseline rates of attrition as the loss of funding for ER-Rs. The typical duration of a Discovery Grant is 5 years, so each year ~20% of the cohort of ER-Rs is under review. We assumed that all established researchers eligible for grant renewal submitted a proposal at the end of their funding period. Our projection model considers that recruitment takes place through successful grant proposals from ER-NHG or ECRs. We adjusted the rates for each group according to their proportional representation in the total population of applicants. Accordingly, our forecasting model conforms to the characteristics of a standard demographic model projecting individual recruitment and attrition [23]. We included stochasticity in our projections by including the annual variability in grant proposal success observed from 2011–2014 for each type of applicant, through 1000 iterations. Note that the forecasting model was parameterized using our detailed dataset (2011–2014) but there were temporal shifts in funding success beyond this time period that could influence our results. Due to lack of data, the model could not be fully parameterized using these additional years but the results of our projections are qualitatively similar across a broad range of hypothetical parameter estimates (D. Murray, *unpubl*.). More importantly, we conducted a sensitivity analysis by varying individual parameters across a range of values and documenting the relative contribution of applicant status to changes in funding rates according to institution size [23, 24].

### Corrective scenarios

We examined the potential for corrective measures to address observed patterns of funding skew and attrition, by projecting the change in the number of grant holders under five different scenarios. Scenario 1 assumed that no measures are used to mitigate institution size bias. Scenario 2 included NSERC’s recent (2015) pilot program of Discovery Development (DD) grants. These modest grants ($10,000 annually for 2 years) are awarded to individuals from small institutions who submitted fundable proposals but still failed to meet the minimum threshold for Discovery Grant support [25]. During the 2015 funding competition NSERC provided 57 DD grants to researchers; this number was used as a corrective measure in each projected year in Scenario 2. We assumed that, after two years, DD recipients would have a Discovery Grant application success rate (i.e., recruitment rate) corresponding with mean success rates (2011–2014) for established researchers (ER-R and ER-NHG combined; this yielded a 6.3% and 12.9% increase in success rate for ER-R and ER-NHG applicants, respectively). Although we expect that most DD grant holders will have scores at the end of the 2-year transition period that more closely reflect those of ER-NHGs who are not currently funded, the outcome of our projection is qualitatively similar across a range of success rates. We assumed that the DD program would be maintained at the same rate through the next decade, spanning the timespan of our projections and two complete grant renewal cycles.

Our third scenario incorporates the recommendation from a 2014 external review of the Discovery Grant program that suggested scores from the three evaluation criteria should be selectively weighted (40% EoR, 40% MoP, 20% HQP) rather than the equal weighting currently given to each [26]. Although NSERC did not adopt this suggestion, we modeled its outcome (Scenario 3) by re-running the projections by funding all grant applications that received a minimum score of “Strong” for both EoR and MoP, and “Moderate” for HQP. Finally, we ran two additional scenarios, where the HQP metric was not considered but rather funding success was determined exclusively by scores from EoR and MoP (Scenario 4) and EoR alone (Scenario 5). In these cases, we used a minimum ‘Strong’ score as the funding threshold. We justify these latter scenarios by the fact that across all combinations of applicant type and institution size, the three evaluation criteria are highly correlated (see below) and, to some degree, they are redundant. Note that the validity of the MoP criterion is further questionable because Discovery Grants are specifically designed to support research programs rather than research projects [19]. There is no formal evaluation of whether research that was proposed in the grant application was actually conducted during the funding period, which reinforces the flexibility of the Discovery Grant program. To summarize, Scenario 1 represents no corrective measures, Scenario 2 makes adjustments that favor established researchers from small institutions, whereas Scenarios 3–5 involve general adjustments to the scoring system that can influence performance by all applicants.

### Ethics statement

Our dataset was provided by NSERC and does not include applicant name, institution name, or other information that would allow the association between funding success and individual or institutional identity. Therefore, the dataset is completely anonymous and consent to participate in the study was not necessary.

## Results and Discussion

**Fig 1:**
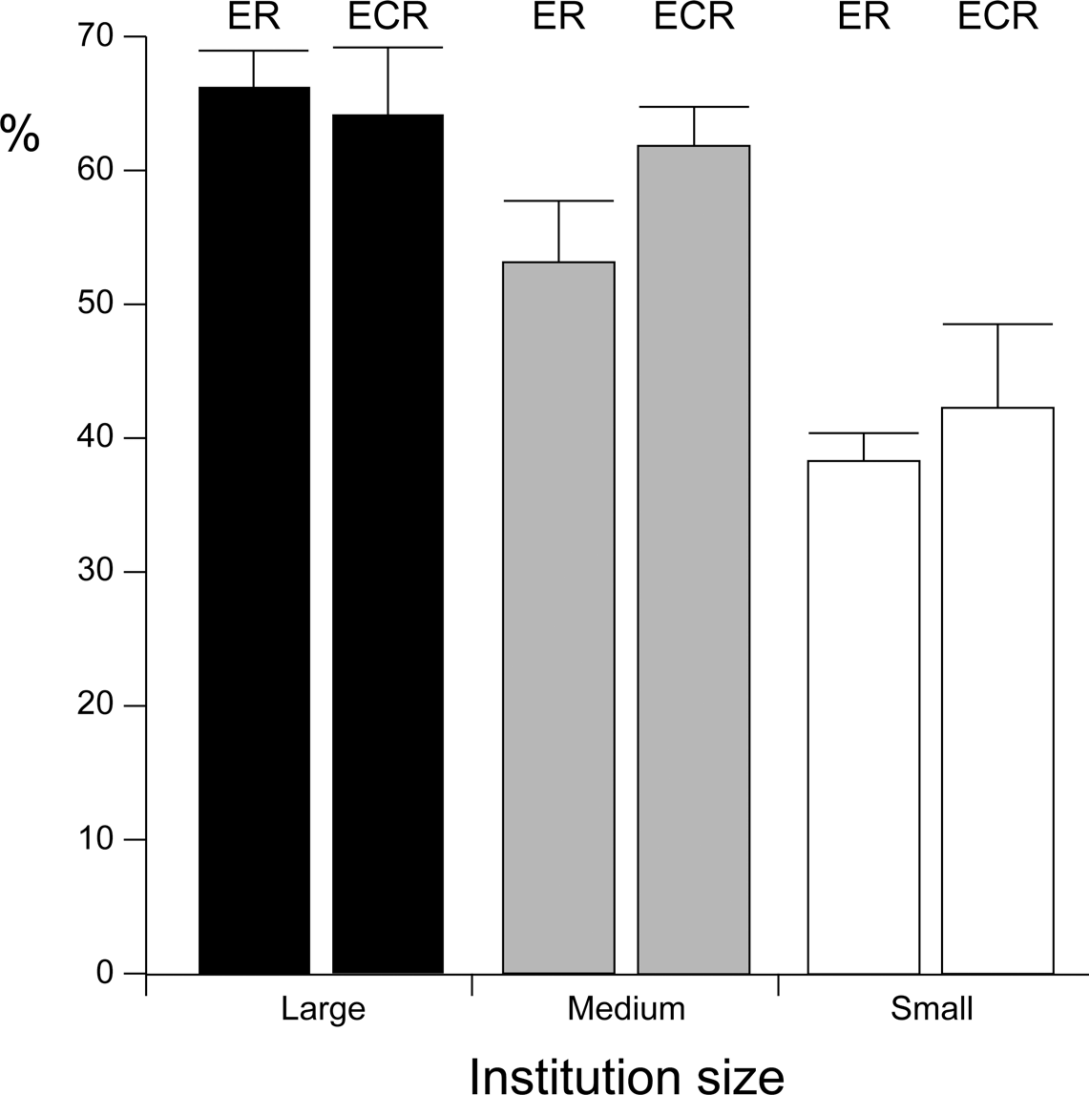
(A) Mean (± SD) percent success of NSERC Discovery Grant applications (2011–2014) relative to institution size and applicant status.

**Fig 2:**
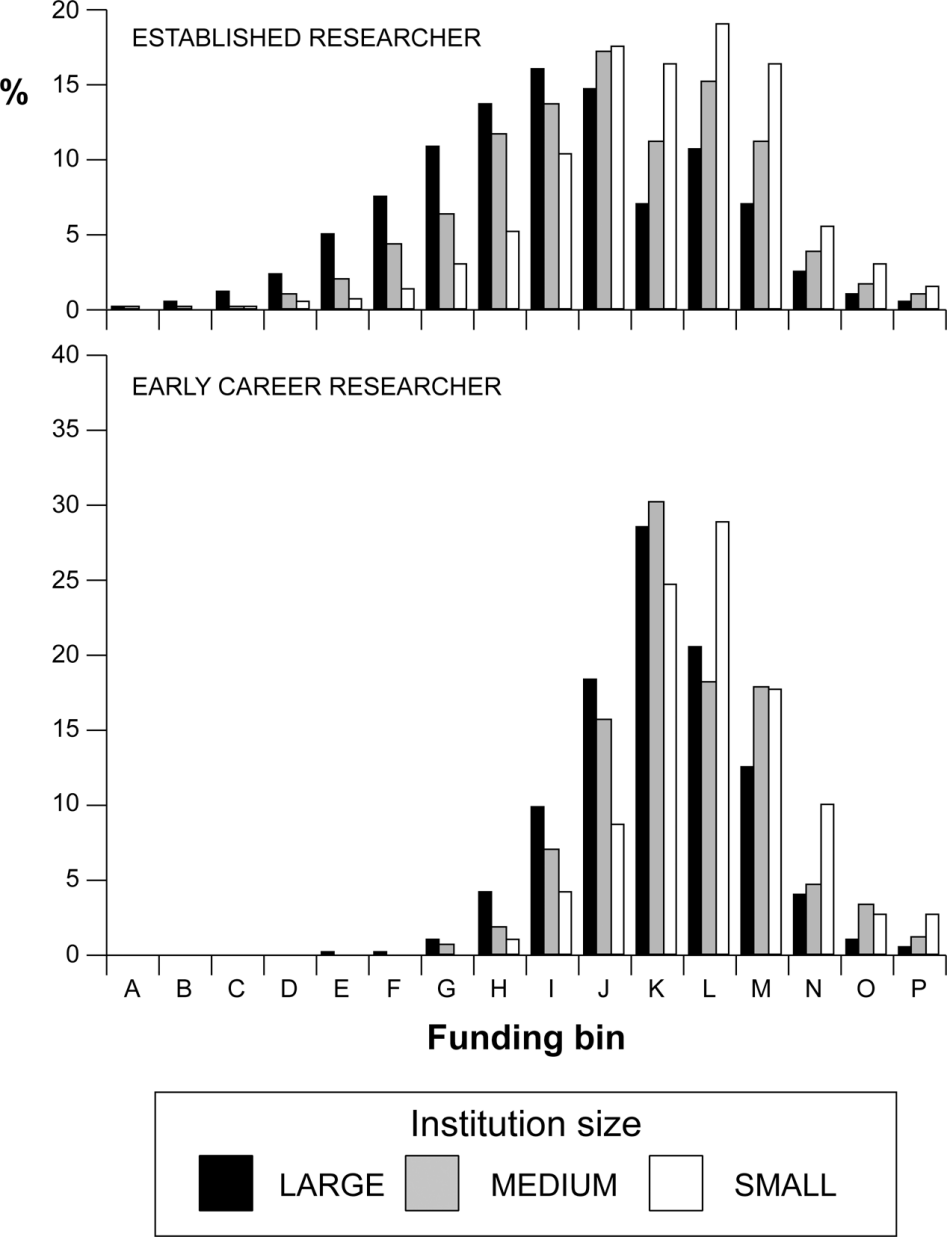
Funding level scores for NSERC Discovery Grant applications (2011–2014) by established researchers and early career researchers, according to institution size. Normally a score of “J” or earlier letter is required for funding.

Our analysis revealed that both funding success and the amount awarded varied with the size of the applicant’s institution. Overall, funding success was 20% and 42% lower for established researchers from medium and small institutions, compared to their counterpart’s at large institutions (Fig 1). For established researchers, the best-fit model for funding success revealed lower probabilities of applicant failure in 2012 and 2014 (compared to the reference year, 2011) and 1.6 or 2.7 times higher chance of failure in comparison with large institutions, for applicants from medium and small universities, respectively (Table 2). Funding success for established researchers holding a Discovery Grant was 6.2 times higher than for their counterparts not currently holding grants. These results were reinforced when the analysis focused on funding level, with even higher odds ratios (Table 2) and strong right skew in funding (Fig 2), thereby showing clear disadvantage for established researchers according to institution size.

**Table 2:** Summary statistics for best-fit models from NSERC Discovery Grant proposal funding success and award outcomes (2011–2014) for established researchers. The variables include years, institution size, and applicant status (established researcher not holding grant (ER-NHG)). The evaluation metrics reflect researcher accomplishments (Excellence of the Researcher, EoR), research proposal (Merit of the Proposal, MoP), and training record and opportunities (High Quality Personnel, HQP).

Measure	2012	2013	2014	Medium	Small	ER-NHG	Pseudo R^2^
Funding awarded	0.825 (0.042)	-	0.741 (0.040)	1.577 (0.089)	2.678 (0.167)	6.205 (0.265)	0.158
Funding bin	0.883 (0.034)	-	-	1.749 (0.078)	2.901 (0.142)	6.841 (0.256)	0.067
Researcher (EoR)	-	-	0.893 (0.036)	2.172 (0.102)	3.496 (0.183)	6.166 (0.244)	0.099
Proposal (MoP)	-	1.136 (0.044)	-	1.443 (0.067)	2.085 (0.106)	4.715 (0.180)	0.068
Training (HQP)	-	-	0.851 (0.034)	1.145 (0.068)	2.343 (0.122)	5.678 (0.225)	0.083

**Notes:** Odds ratios (± SE) for variables in best-fit models, with reference values (Year 2011, Large universities, ER-R). All individual variables retained in models are significant (*P*<0.050). Higher odds ratios indicate increased odds of poorer success. Sample size is 11, 700.

**Fig 3:**
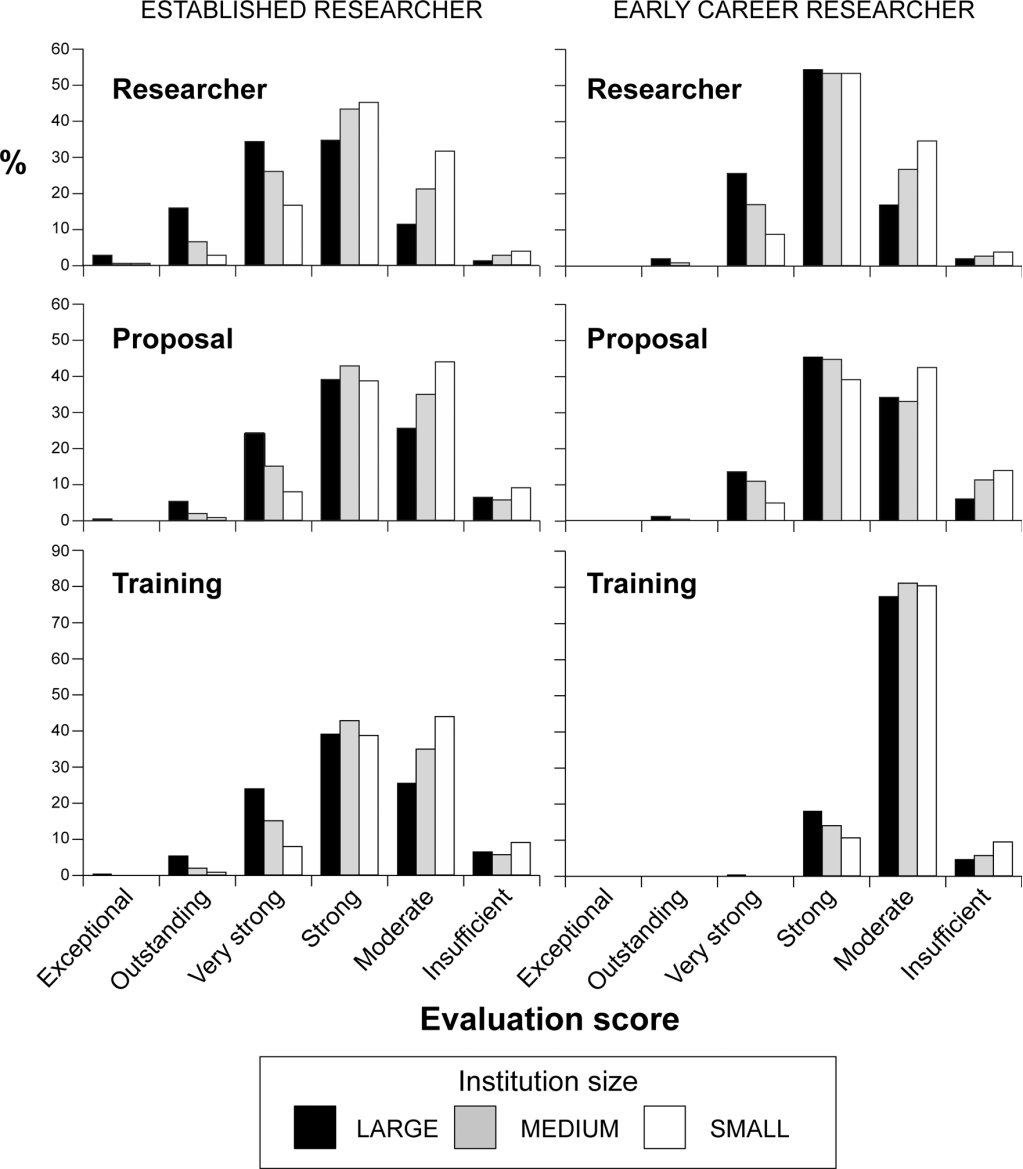
Evaluation scores for NSERC Discovery Grant applications relative to institution size (2011–2014). (A) Excellence of the Researcher for established researchers. (B) Excellence of the Researcher for early career researchers. (C) Merit of the Proposal for established researchers. (D) Merit of the Proposal for early career researchers. (E) Contribution to the Training of High Quality Personnel for established researchers. (F) Contribution to the Training of High Quality Personnel for early career researchers.

Overall, the observed shift in funding level (Fig 2) translates to 20% and 19% smaller grant sizes being received by ER-Rs at medium and small institutions, respectively, and 15% and 20% smaller grants for ER-NHGs at medium and small schools, respectively [18]. Further, when we examined established researcher evaluation scores according to the three evaluation criteria, we found them to be highly correlated (Kendall’s Rank Correlation, all tau>0.369, *P*<0.001), as evidenced by the consistently higher odds ratios among evaluation criteria for applicants from small schools (Table 2). Indeed, there was a consistent right skew in evaluation scores for all criteria, in relation to institution size (Fig 3).

For ECRs, overall funding success was 4% and 34% lower for applicants from medium and small institutions, respectively, compared to their counterpart’s at large institutions. For ECRs, the observed lower funding success at small schools (Fig 1) translated to a 2.4 times higher failure rate compared to applicants from large institutions (Table 3). Funding levels were higher for applicants from large schools compared to medium and small schools (Table 3), translating to 6% and 9% smaller grant sizes being received by ECRs at medium and small institutions, respectively [18]. As was the case with established researchers, for ECRs the three evaluation criteria were correlated (all tau>0.133, *P*<0.001), and there was a right skew for each criterion among applicants from small institutions (Fig 3).

**Table 3:** Summary statistics for best-fit models from NSERC Discovery Grant proposal funding success and award outcomes (2011–2014) for Early Career Researchers. The evaluation metrics reflect researcher accomplishments (Excellence of the Researcher, EoR), research proposal (Merit of the Proposal, MoP), and training record and opportunities (High Quality Personnel, HQP).

Measure	2012	2013	2014	Medium	Small	Pseudo R^2^
Funding awarded	-	-	0.755 (0.088)	-	2.482 (0.315)	0.024
Funding bin	0.798 (0.077)	-	-	1.532 (0.182)	2.266 (0.300)	0.012
Researcher (EoR)	0.766 (0.079)	-	-	1.819 (0.235)	3.014 (0.369)	0.023
Proposal (MoP)	-	-	-	1.287 (0.161)	2.089 (0.247)	0.009
Training (HQP)	1.797 (0.279)	3.405 (0.562)	2.236 (0.360)	1.436 (0.236)	2.263 (0.372)	0.034

**Notes:** Odds ratios (± SE) for variables in best-fit models, with reference values (Year 2011, Large universities). All individual variables retained in models are significant (*P*<0.050). Higher odds ratios indicate increased odds of poorer success. Sample size is 1826.

### Differential merit or systemic bias?

**Fig 4:**
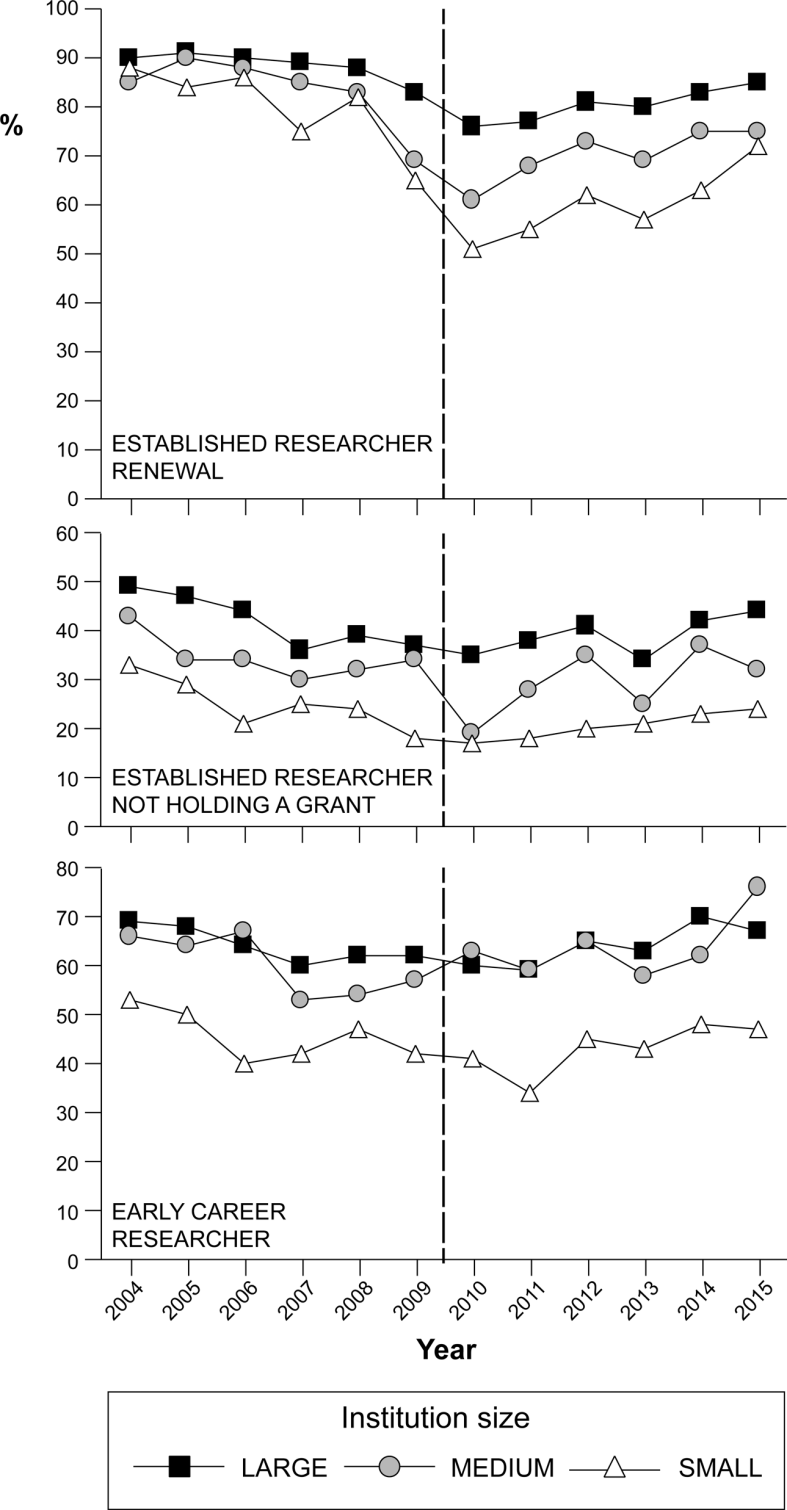
Discovery Grant success rates 2004–2015. (A) Established researchers currently seeking Discovery Grant renewal (ER-R). (B) Established researchers currently not holding a Discovery Grant (ER-NHG). (C) Early career researchers (ECR). The dashed line reflects the 2009–2010 adoption of a new grant application evaluation system.

Our longer-term dataset revealed that lower scoring at small institutions has persisted at least since 2004 (Fig 4) and, collectively, our results call into question the source of the skew observed in Discovery Grant proposal evaluation scores. In general, applicants from medium and small institutions may receive lower scores simply because they have weaker research records, perhaps as a result of higher teaching or administrative commitments compared to individuals from larger schools. Indeed, establishment of successful research programs is closely linked to the availability of time to conduct research [27, 28], which may be more limited at smaller institutions. Researchers at small schools may also have fewer local collaborators and research-related resources than their counterparts at larger schools [29]. Given these disparities, observed funding skew may be a consequence of the context in which applicants find themselves rather than emerging from a systemic bias during grant proposal evaluation. However, two lines of evidence suggest that the observed disparity is at least partly caused by bias.

First, both established researchers and ECRs from small schools have lower funding success, indicating that applications from these institutions are consistently under-valued. Notably, the magnitude of difference in the success rates according to school size is surprisingly consistent, irrespective of applicant experience level (Fig 1, Tables 2 and 3). However, ECRs are recently appointed to their first faculty position and their record mainly represents achievements from their student and post-doctoral training at institutions other than where they are currently employed. Indeed, two of the scoring criteria, researcher excellence (EoR) and personnel training (HQP), should be shaped by standardized measures like number of publications, quality of publications, number of graduate students, and number of graduate student publications (S1 Table). These measures should be independent from quality of the written research proposal (MoP), but as we demonstrated earlier, all evaluation metrics are correlated. Further, given the contemporary job market and the glut of PhDs [30, 31], it is unlikely that small schools systematically hire weaker researchers to fill tenure-track positions, or that elite ECRs accept offers only from large institutions. Although in some cases ECRs from small schools might lack the institutional resources necessary to prepare a strong Discovery Grant research proposal, this seems improbable as a source of skew because most ECR research proposals support programs that expand upon their previous research experience, which should not be notably weaker for ECRs from smaller schools. Thus, the overall consistency in skew between established researchers and ECRs suggests a skew resulting from evaluation bias.

Second, the consistently lower evaluation scores among ECRs from small schools imply that these applicants are weaker in all facets of research and training (Table 3). However, the right skew in scores for personnel training by small-school applicants (Fig 3) is questionable given that all ECRs, regardless of current institution size, have limited experience in student mentoring. Indeed, the right skew for ECR training is significant for applicants from both medium and small institutions (Table 3). However, it is unclear how such skew could arise in the absence of systemic bias, when virtually all ECRs have experienced little previous opportunity to train personnel.

### Long-term consequences

**Fig 5:**
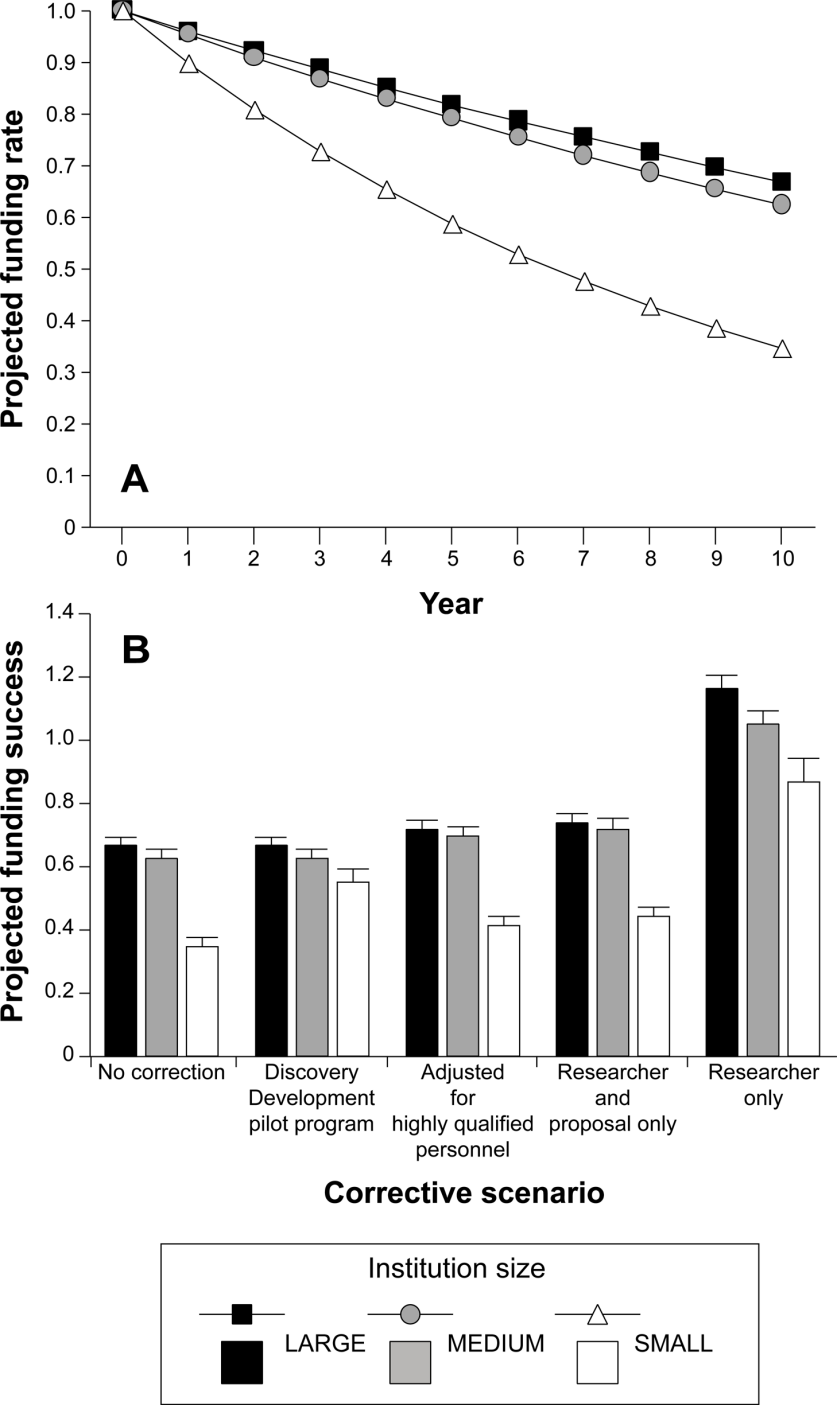
(A) Projected 10-year mean change in NSERC Discovery Grant funding at Canadian universities assuming no corrective measures to address bias related to institution size. (B) Projected mean (± SD) 10-year change in NSERC Discovery Grant funding at universities according to different bias-corrective measures, relative to current funding rates. Values less than 1 represent attrition in funded researchers.

How do these patterns translate into future science research funding at small schools? When current grant proposal success and funding scores are projected forward, funding support declines at all institutions, but especially so at small schools (Fig 5). If left unabated, our projections show that during the next decade, the number of funded researchers will decline by one third at large and medium-sized schools, and by two thirds at small ones. This outcome is due to the combined effects of higher attrition in funding for established researchers who currently hold a Discovery Grant, and lower recruitment into funded bins for ECR’s and established researchers currently not holding a Discovery Grant (Fig 2). Our sensitivity analysis of model parameter estimates shows that lack of successful recruitment of ECRs into the ranks of funded researchers is important at small institutions, albeit proportionally less than at larger institutions (Table 4). Further, we note that these estimates are likely conservative because they do not account for the anticipated negative feedback associated with diminished success rates and loss of propitious research environments at small schools. Accordingly, as fewer researchers receive Discovery Grant funding at smaller institutions, the loss of both funded collaborators and a critical mass of researchers required to conduct successful research, will be compounded.

**Table 4:** Sensitivity analysis of applicant type contributions for projected Discovery Grant funding success relative to institution size. Applicant types are established researchers seeking grant renewal (ER-R), established researchers not currently holding a grant (ER-NHG) and early career researchers (ECR).

Applicant type	Large	Medium	Small
ER-R	0.238	0.25	0.368
ER-NHG	0.672	0.698	0.558
ECR	0.65	0.618	0.57

**Notes:** The table reports the proportional reduction in funding success rates over 10 years resulting from complete removal of the contribution of individual applicant types. Projections assume that no corrective measures are applied.

The disadvantage of ECRs in terms of both grant proposal funding success and funding levels (Table 3), is particularly troublesome. Small institutions, if they are to maintain a viable research capacity, require a comparable rate of successful recruitment of ECRs into the Discovery Grant program. Their ability to conduct research and training will be further eroded unless national research councils reverse contemporary trends that concentrate grant funding in the hands of select researchers and elite universities [32, 33].

An important additional observation emerging from our data is that there exists a consistently high rate of attrition among currently-funded established researchers (ER-R) who are unsuccessful in renewing their Discovery Grant at the end of a 5-year grant cycle (Fig 4). This trend is especially prominent at small institutions, where ER-Rs have a disproportionately strong influence on the decline in institutional Discovery Grant success (Table 4). This finding is especially problematic for programs such as NSERC Discovery Grants, which are intended to support research programs rather than specific research projects [19].

Researchers need consistent access to funds in support of research and training opportunities in order to maintain productivity and continuity in a novel research program. These opportunities disappear when funding is lost mid-career. A high proportion of established researchers lacking a Discovery Grant (ER-NHG) in the applicant pool (2009–2015 range: 25.8–33.8%, see 18) as well as their low funding rate (see Fig 4) mainly reflects low recruitment of former ER-Rs who have lost their Discovery Grants. We interpret this as a direct failure to achieve the Discovery Grant program’s fundamental objective of supporting “ongoing programs of research (with long-term goals) rather than a single short-term project or collection of projects” [34]. Indeed, researchers who initially receive a Discovery Grant only to lose funding later in their career, represent inefficiency in NSERC research spending; a more effective funding model would either increase the likelihood of continuity of Discovery Grant funding through the duration of an applicant’s research career, or else better identify the group of ECRs who are least likely to receive a grant renewal, and not fund their initial funding request. The large cohort of ER-Rs from small schools, who become ER-NHGs after their Discovery Grant renewal is unsuccessful, aggravates the science funding problem facing smaller institutions.

### Mitigating funding skew

The integrity of any national research council depends on the premise that successful funding is merit-based, and it is thus the responsibility of research councils to invoke measures to mitigate unwanted evaluation skew. NSERC began to address this issue in 2015 with its Discovery Development (DD) program that provides bridge funding to applicants at small schools who narrowly missed the minimum criteria for a normal Discovery Grant. Fifty-seven established researchers from small schools each received $20,000 over two years. The amount is less than 30% of the 2015 mean annual funding for successful proposals [18] and surely is insufficient to operate a viable research program during the transition period. Even if matched by internal grants, DD recipients will receive substantially less research funding than the national mean grant. Further, based on past success rates for established researchers from small schools (Fig 1), it is unlikely that the DD program will dramatically reverse the pattern of low funding success. In particular, the largest skew in scores for applicants from medium and small schools is due to the researcher excellence (EoR) criterion (Tables 2 and 3, Fig 3), which is unlikely to be reversed over the 2-year span of the DD award. Although the DD program acknowledges and aims to correct skew in grant proposal funding success, it fails to address the root causes of lower Discovery Grant funding among applicants from small schools. Thus, it is difficult to envision how short-term initiatives such as DD awards will remedy evaluation skew that systematically disfavours applicants from small schools. Instead, research funds would be more wisely invested if they provided sufficient support for all qualified researchers to maintain viable and productive long-term research and training programs.

NSERC Discovery Grants may be adjusted through other corrective measures including lower weighting for personnel training [26]. This action may be especially appropriate for new researchers given that the training criterion is largely non-informative for ECRs in the Discovery Grant competition (Fig 2). However, our projections show that such a change would not substantially alter the observed skew in funding success (Fig 5). Other corrections, including eliminating scores for the research proposal, continue to show persistent differential funding rates according to institution size (Fig 5).

### Implications to science funding policy

Systemic evaluation skew is increasingly evident among other national research councils [31–33], although in most cases it remains unclear to what extent such skew is attributable to differential merit and/or actual evaluation bias. Regardless of its source, this skew is challenging to address but there may be measures that can better mitigate its effects than current solutions [31]. First, one must be clear on the source of the problem that may often be rooted in limited national funds for research or shifting funding priorities toward commercial development and innovation.

Second, it seems evident that, in the case of NSERC Discovery Grants, early career researchers should not be evaluated using the same scoring system as established researchers. Based on our analysis, either a different HQP training criterion should be developed, or the criterion should be dropped altogether for ECRs. An alternative is to follow the lead of other research councils that offer starting grants restricted to early career applicants [35–37]. Evaluation criteria for these programs can then be tailored to reflect the best indicators of future research success.

Third, applicant identity and institutional affiliation are cornerstones for assessing researcher excellence (and perhaps personnel training), meaning that evaluation bias may be difficult to avoid unless opportunities for subjective assessment are minimized. This may be facilitated through greater reliance on bibliometric or axiomatic indices [38–40], especially those emphasizing recent contributions or criteria that are prioritized by the research council or funding competition. These metrics should be tabulated by the research council rather than by individual grant proposal reviewers and selection committee members, as is currently the approach with Discovery Grants (see S1 Table). In fact, standard bibliometric approaches have been adopted by other research councils, including in Canada [41], and thus reasonably could be integrated into NSERC Discovery Grant proposal evaluations. These metrics should complement other measures of researcher productivity, quality, and novelty that may be prioritized differentially by individual research councils. For instance, some research councils may recognize contributions to scholarship, the profession, and training as being particularly important, and these can be quantified and integrated directly into the evaluation score.

Fourth, the research proposal component of the grant application necessarily must be evaluated by expert reviewers and selection committees rather than through bibliometric approaches, but this can be accomplished via blind review. Such an approach will separate an applicant’s institutional affiliation from the proposed work, and thereby should limit spurious correlation between scores for researcher excellence and proposal merit, even in a small research community where reviewers may be familiar with individual applicants [42, 43]. Blind review is becoming increasingly common as a means of ensuring objectivity for manuscripts under consideration for publication [44, 45], although a potential drawback of blind review of research grant proposals is the loss of context between applicants’ experience and their ability to successfully conduct the proposed work. However, this concern does highlight the questionable value of research proposals in the Discovery Grant context, given that the three evaluation criteria yield correlated scores, and that there is no direct accountability between research that is proposed in a grant application and that which is actually conducted using granted funds. Importantly, as new funding evaluation systems are developed and launched, research councils must be explicit and transparent by using metrics that are fair, relevant, and well-tested, thereby allowing researchers to confidently tailor their research activities and funding proposals for best success. For example, as it stands the Discovery Grant program lacks clarity in terms of both the relative importance of quantity versus quality of publications when evaluating researcher performance, and the role of trainee degree type (i.e., B.Sc., M.Sc., Ph.D) and post-graduation employment activity, when assessing quality of the training environment. These are important considerations when developing any robust and informative grant evaluation system. Regardless, whatever mechanisms are chosen to improve funding equity, they should remove the institutional context of research applications and/or acknowledge evaluation skews and develop policies and procedures that will effectively mitigate their consequences.

### Institutional responsibilities

It is important to emphasize that even with earnest reform of grant proposal evaluation criteria and procedures, there will likely remain tangible disadvantages to research funding success and productivity at small universities. Some of these residual disadvantages may reflect true differences in applicant merit, and some small institutions probably contribute to low grant proposal funding success by failing to provide highly competitive research environments for their faculty. In response, small institutions can be pro-active by developing policies that better reflect contemporary challenges and realities in an increasingly competitive and cash-limited research environment.

First, new researchers should be recognized as needing adequate institutional support to establish productive research programs. While the process of job negotiation between university administration and potential new faculty often can be adversarial, administrators have a responsibility to provide startup funds, reduced teaching loads, and necessary space and support that will set a strong foundation for researcher success during the initial phases of their careers. Some institutions have adopted peer-mentoring policies for new researchers as an added level of support which can assist with grant proposal or manuscript editing, research direction, and helping balance conflicting time demands [46, 47].

Second, established researchers at small schools should receive the necessary institutional support to maintain research success. Examples include adjusted teaching loads, improved administrative support for research, and reduced institutional duties such that researchers have sufficient time to conduct research and publish papers, write competitive grant proposals, and train and manage personnel [48, 49]. Some institutions hire teaching-only faculty who can provide some teaching relief, thereby allowing researchers to increase investment in research-related endeavors. Alternatively, small schools may provide flexibility in teaching and research responsibilities according to faculty performance or career priorities [50]. In many cases, robust graduate student funding through undergraduate teaching or research assistantships can greatly benefit researcher capacity to support active research programs; our data (Table 1) show that small institutions have proportionally fewer graduate students, and by inference, lower research capacity [48, 49]. Intramural bridge funding, for established researchers who recently lost research grants, may increase the likelihood that extramural support eventually can be regained.

Third, small schools should recognize that in the present funding and student recruitment climate, it may no longer be possible to mount successful research in all traditional disciplines. Rather, small institutions need to select strategic areas for research emphasis and development, and capitalize on synergies and economies of scale by supporting core groups of researchers who can collaboratively establish strong and dynamic research programs that will be competitive with those at larger schools. During such transition, comparable support to productive researchers who fall outside of changing strategic initiatives should be maintained. It follows that small institutions need to be creative and nimble in their approach to research and graduate training, such as by adopting accelerated graduate degree programs to facilitate more efficient personnel training, or refining existing programs to keep pace with emerging and dynamic disciplines, new technologies, and the knowledge requirements of changing societies. Importantly, small institutions need to organize and speak with a united voice in support of research at small schools, including fair and equitable science funding that recognizes varying research contexts among institutions. Large institutions have been particularly effective in these pursuits through efforts that enable unified positions, successful lobbying of governments, and active strategic planning and institutional restructuring. Ultimately, adopting similar measures will benefit small institutions by allowing them to better profile and position themselves in a highly competitive and dynamic science-funding environment.

## Conclusion

Public support for science is an important trust and public research funds must be distributed appropriately and without bias, in order to fully advance knowledge and thereby benefit societal needs and expectations. All applicants must have equal opportunity for success through an evaluation process that is based upon future research prospects, capabilities, and accomplishments rather than the applicant’s seniority, celebrity, or institutional affiliation. Such a diversified approach promotes breadth in a nation’s science training environment, scientific accomplishments, and overall productivity [1–3]. Ultimately, elimination of systemic skew in grant proposal evaluation and funding is the responsibility of individual research councils and will improve a nation’s ability to maintain scientific excellence while attracting, training, and retaining talented scholars.

## Supporting Information

S1 Table S1 Table presents the qualitative criteria used by grant proposal reviewers and selection committees to evaluate NSERC Discovery Grant proposals, see: http://www.nserc-crsng.gc.ca/_doc/Professors-Professeurs/DG_Merit_Indicators_eng.pdf (accessed August 20, 2015). (DOCX)
