# Supplementary material for: Bias in Research Grant Evaluation Has Dire Consequences for Small Universities
Source: PLoS One. 2016 Jun 3;11(6):e0155876. doi: 10.1371/journal.pone.0155876 (PMC4892638; doi:10.1371/journal.pone.0155876)
Supplement: S1 Table — (DOCX) [file pone.0155876.s001.docx]

**Table S1. Discovery Grant merit indicators.** Source: <http://www.nserc-crsng.gc.ca/_doc/Professors-Professeurs/DG_Merit_Indicators_eng.pdf> (August 2015)

|  | **Exceptional** | **Outstanding** | **Very Strong** | **Strong** | **Moderate** | **Insufficient** |
| --- | --- | --- | --- | --- | --- | --- |
| **Excellence of the Researcher** | Acknowledged as a **leader** who has continued to make, over the last six years, **influential accomplishments** at the highest level of quality, impact and/or importance to a **broad community**. | The accomplishments presented in the application were deemed to be **far superior** in quality, impact and/or importance to a **broad community.** | The accomplishments presented in the application were deemed to be of **superior** quality, impact and/or importance. | The accomplishments presented in the application we deemed to be **solid** in their quality, impact and/or importance. | The accomplishments presented in the application were deemed to be of **reasonable** quality, impact and/or importance. | The accomplishments presented in the application were deemed to be **below an acceptable** **level** of quality, impact and/or importance. |
| **Merit of the Proposal** | Proposed research program is clearly presented, is **extremely original and innovative** and is highly **likely to have impact by leading to groundbreaking advances** in the area and/or **leading to a technology or policy** that addresses socio-economic or environmental needs. **Long-term vision** and **short-term objectives** are **clearly defined**. The methodology is **clearly defined and appropriate.** The proposal and budget **clearly demonstrate** how the research activities to be supported are distinct from and complement those funded by other sources. | Proposed research program is clearly presented, is **highly original and innovative** and **is likely to have impact by contributing to groundbreaking advances** in the area, and/or **leading to a technology or policy** that addresses socio-economic or environmental needs. **Long-term goals are clearly defined and short-term objectives are well planned.** The methodology is **clearly described and appropriate.** The proposal and budget clearly **demonstrate** how the research activities to be supported are distinct from and complement those funded by other sources. | Proposed research program is clearly presented, is **original and innovative** and **is likely to have impact by leading to advancements** and/or addressing socio-economic or environmental needs. **Long-term goals are defined and short-term objectives are planned.** The methodology is **clearly described and appropriate.** The proposal and budget **demonstrate** how the research activities to be supported are distinct from and complement those funded by other sources. | Proposed research program is clearly presented, is original and innovative and is likely to have impact and/or address socio-economic or environmental needs. Long-term goals and short-term objectives are clearly described. The methodology is described and appropriate. The proposal and budget demonstrate how the research activities to be supported are distinct from and complement those funded by other sources. | Proposed research program is clearly presented, has **original and innovative** aspects and is **likely to have impact** and/or address socio-economic or environmental needs. **Long-term goals and short-term objectives are clearly described.** The methodology is **partially described** **and/or appropriate.** The proposal and budget somewhat **demonstrate** how the research activities to be supported are distinct from and complement those funded by other sources. | Proposed research program, as presented **lacks clarity,** and/or is **of limited originality and innovation. Objectives are not clearly described** and/or likely not attainable. Methodology is **not clearly described and/or appropriate.** The proposal and budget **do not clearly demonstrate** how the research activities to be supported are distinct from and complement those funded by other sources. |
| **Training of HQP** | Training record is **at the highest level,** with HQP contributing **to top quality research.** **Most** HQP move on to positions that require **highly desired** **skills**, obtained through training received. Research plans for trainees are **appropriate and clearly defined.** HQP success is **highly likely.** | Training record is **far superior** to other applicants, with HQP contributing to high-**quality research.** **Most** HQP move on to positions that require **highly desired skills,** obtained through training received. Research plans for trainees are **appropriate and clearly defined.** HQP **success highly likely.** | Training record is **superior** to other applicants, with HQP contributing to **quality, original research.** **Many** HQP move on to appropriate positions that require **desired skills,** obtained through training received. Research plans for trainees are **appropriate and clearly-described.** **HQP success is likely.** | Training record compares **favourably** with other applicants. HQP **generally** move on to positions that require **desired skills**, obtained through training received. Research plans for trainees are **appropriate and described.** HQP **success is likely.** | Training record is acceptable but may be modest relative to other applicants. **Some** HQP move on to programs or positions that require **desired skills,** obtained through training received. Plans for trainees are **described** and **should contribute to HQP success.** | Training record is **below an acceptable level** relative to other applicants. HQP do not, in general, move on to positions that require skills obtained through training received. Plans for trainees are **not appropriate** or are **not described** with enough **information to predict likelihood of HQP success.** |
